# 5′-Cytimidine Monophosphate Ameliorates H_2_O_2_-Induced Muscular Atrophy in C2C12 Myotubes by Activating IRS-1/Akt/S6K Pathway

**DOI:** 10.3390/antiox13020249

**Published:** 2024-02-19

**Authors:** Xin Wu, Na Zhu, Lixia He, Meihong Xu, Yong Li

**Affiliations:** 1 Department of Nutrition and Food Hygiene, School of Public Health, Peking University Health Science Center, Beijing 100191, China; wuxin12@bjmu.edu.cn; 2 Beijing Key Laboratory of Protein Posttranslational Modifications and Cell Function, Department of Biochemistry and Molecular Biology, School of Basic Medical Science, Peking University Health Science Center, Beijing 100191, China; 3 Department of Nutrition and Food Hygiene, College of Public Health, Inner Mongolia Medical University, Hohhot 010059, China; 20220828@immu.edu.cn; 4 Division of Molecular and Cellular Oncology, Dana-Farber Cancer Institute, Brigham and Women’s Hospital, Harvard Medical School, Boston, MA 02115, USA; lixia_he@dfci.harvard.edu; 5 Beijing Key Laboratory of Toxicological Research and Risk Assessment for Food Safety, Peking University Health Science Center, Beijing 100191, China

**Keywords:** 5′-cytimidine monophosphate, sarcopenia, muscular atrophy, aging

## Abstract

Age-related muscle atrophy (sarcopenia), characterized by reduced skeletal muscle mass and muscle strength, is becoming increasingly prevalent worldwide, which is especially true for older people, and can seriously damage health and quality of life in older adults. This study aims to investigate the beneficial effects of 5′-cytimidine monophosphate (CMP) on H_2_O_2_-induced muscular atrophy in C2C12 myotubes. C2C12 myotubes were treated with H_2_O_2_ in the presence and absence of CMP and the changes in the anti-oxidation, mitochondrial functions, and expression of sarcopenia-related proteins were observed. Immunofluorescence analysis showed that CMP significantly increased the diameter of myotubes. We found that CMP could increase the activity of antioxidant enzymes and improve mitochondrial dysfunction, as well as reduce inflammatory cytokine levels associated with sarcopenia. RNA-seq analysis showed that CMP could relieve insulin resistance and promote protein digestion and absorption. Western blot analysis further confirmed that CMP could promote the activation of the IRS-1/Akt/S6K signaling pathway and decrease the expression of MuRF1 and Atrogin-1, which are important markers of muscle atrophy. The above results suggest that CMP protects myotubes from H_2_O_2_-induced atrophy and that its potential mechanism is associated with activating the IRS-1/Akt/S6K pathway to promote protein synthesis by improving mitochondrial dysfunction and insulin resistance. These results indicate that CMP can improve aging-related sarcopenia.

## 1. Introduction

Sarcopenia, the degenerative loss of skeletal muscle mass, quality, and strength, severely harms the health of older adults, leading to increased clinically adverse events such as falls, fractures, physical disability, and mortality. Muscular atrophy is a characteristic of sarcopenia. Muscle atrophy essentially involves a reduction in muscle fiber diameter, resulting in decreased mass and weakened function. Sarcopenia has become a serious global health problem [1]. Muscle mass decreases by 1–2% every year in people over 50 years of age, while muscle strength declines by 1.5% every year between 50 and 60 years of age and by 3% in people over 60 years of age [2]. With the increase in age, the incidence of sarcopenia gradually increases. The incidence of sarcopenia is 5–13% in the elderly over 65 years old and as high as 50–60% in the elderly over 80 years old [3]. Sarcopenia increased morbidity and mortality from chronic disease, falls, extended hospital stays, and loss of ability to live independently. Sarcopenia is associated with huge personal and social financial cost [4,5], and, for instance, the total cost of sarcopenia is about USD 18.4 billion for the American healthcare system [6].

Sarcopenia, with a high prevalence and posing serious health hazard, still lacks a pharmacological therapy. It is very obvious that therapies are desperately needed. Identifying cost-effective interventions to maintain muscle mass, muscle strength, and physical performance in older adults is a major public health challenge. Resistance training and diet intervention are currently recommended as the gold standard for sarcopenia treatment. Resistance training is difficult to achieve for the elderly, especially those who are frail or bedridden for a long time. Therefore, it is of great significance to find effective diet intervention strategies for sarcopenia based on a deep understanding of the pathogenesis of sarcopenia.

The pathogenesis of sarcopenia is not completely understood. Studies show that sarcopenia is mainly related to impaired protein metabolism in diverse physiological and pathophysiological conditions [7]. This is mainly a result of the imbalance between skeletal muscle protein synthesis and degradation, which triggers muscle atrophy and even results in atrophy. Metabolic factors can lead to this imbalance, including changes in anabolic hormone levels, catabolic stimulation because of inflammation or disease, insufficient physical activity, and nutritional factors such as inadequate protein intake [8]. In addition, a variety of intracellular changes are related to it, involving protein synthesis regulation, protease activation, ubiquitin binding, and autophagy [9]. Insulin and insulin-like growth factor 1 (IGF-1) are powerful anabolic factors that play an important role in maintaining body and muscle growth. Some studies found that the beneficial effects of IGF-1 mostly relied on the activation of the PI3K/Akt pathway, which promoted protein synthesis and blunted protein degradation [10,11,12]. Since insulin activates the PI3K/Akt pathway, analogously to IGF-1, insulin resistance plays a consistent role in muscle atrophy [13]. Insulin receptor substrates (IRSs), as downstream signaling components of insulin receptors, are key players in insulin signal transduction [14]. Insulin recruits and activates the IRS and activates the downstream Akt cascade [15]. Moreover, the Akt-mTOR-p70s6k cascade is a critical pathway regulating protein synthesis [16]. Akt activates S6 kinase 1 (S6K1) via mammalian target of rapamycin (mTOR), which contributes to increased protein synthesis [16]. Many studies have shown that the p70 ribosomal protein S6 kinase (p70s6k) played a critical role in regulating protein synthesis and muscle growth [16].

Mitochondria not only play an important role in the production of cellular energy but also in regulating reactive oxygen species (ROS) production and antioxidant defense against oxidative stress, a major contributor to sarcopenia. The production of H_2_O_2_ from mitochondria increases with age [17]. An increase in H_2_O_2_ concentration can cause atrophy in cell culture models [18]. Time course analysis showed that this increase occurred before skeletal muscle atrophy [19], which suggests that it might be an important reason for the occurrence of sarcopenia. Therefore, to better simulate skeletal muscle aging resulting from mitochondrial dysfunction and oxidative stress, we induced muscular atrophy in C2C12 cells using H_2_O_2_. Mitochondrial dysfunction and oxidative stress are closely related to sarcopenia [20]. Mitochondria is the main source of ROS in muscle tissue. Increases in ROS can inhibit phosphorylation of Akt, mTOR, and the downstream mTOR targets p70s6k and 4E-BP1 [21]. In addition, ROS can control the redox signaling pathway in muscle fibers, and it significantly reduce the number and function of mitochondria in skeletal muscle. Moreover, ROS can decrease skeletal muscle protein synthesis and promote protein hydrolysis [22]. Studies have found that inflammatory cytokines (such as CRP, TNF-α, IL-6, etc.) played an important role in the decline in muscle strength [23]. It was identified that a high level of TNF-α expression in vivo could inhibit the Akt/mTOR pathway and lead to an increase in muscle protein catabolism and also activate the ubiquitin–proteasome pathway to promote muscle proteolysis [24]. In addition, the phosphorylation of S6K1 decreased in IL-6-infused muscles [25]. Therefore, ROS and inflammation can lead to anabolic resistance in sarcopenia.

Nucleotides are an important substance that determines the biological characteristics and protein structure and function in the somatic cells of organisms. They control the growth, development, reproduction, and inheritance of organisms. Nucleotides are the basic units of nucleic acid macromolecules, involving cytidine, guanine, thymine, thymine, and uracil. In recent years, studies have found that in specific physiological conditions, exogenous nucleotides were an indispensable component of nourishment. When the body was hungry, experiencing liver or immune challenge, or under the condition of rapid growth or recession, exogenous nucleotides could quickly enter the various kinds of organization and be absorbed by the body to utilize [26,27]. Nucleotides may have the potential to ameliorate sarcopenia. It has been found that tissue utilization of nucleotides appears to be tissue dependent, with cells with high energy requirements (e.g., skeletal muscle, brain) and/or cells with vigorous DNA replication (e.g., immune cells, intestinal cells) requiring more nucleotides [28]. A previous study found that healthy men supplemented with dietary nucleotides had significantly delayed exhaustion time [29]. Exogenous nucleotides may promote the repair of mitochondrial DNA damage caused by strenuous exercise [30]. Furthermore, cytidine 5′-monophosphate was found to be decreased in the skeletal muscle of old mice in comparison with that of young mice, which suggests that CMP supplementation might have a positive effect on sarcopenia [31]. The study by Nakagawara et al. also showed that 5′-CMP and 5′-UMP could promote myogenic differentiation and mitochondrial biogenesis in C2C12 myotubes [32]. Therefore, this research was performed to investigate the possible ameliorative effect of CMP against hydrogen peroxide (H_2_O_2_)-induced muscular atrophy in C2C12 cells and the underlying mechanism.

## 2. Materials and Methods

### 2.1. Chemicals

The 5′-cytimidine monophosphate used in our experiment was supplied by HAINAN SHUANGDI ZHEN-AO LIFE SCIENCE RESEARCH CENTER Co., Ltd. (Baoting, Hainan, China).

### 2.2. Cell Culture and Treatments

C2C12 (a myoblastic cell line) cells were purchased from Zhejiang Meisen Cell Technology Co., Ltd. (Hangzhou, China). The C2C12 cells were first cultured in growth medium containing Dulbecco’s Modified Eagle’s Medium (DMEM) (GIBCO, Grand Island, NE, USA) supplement with 1% penicillin/streptomycin (Coolaber, Beijing, China) and 10% fetal bovine serum (Zhong Qiao Xin Zhou Biotechnology Co. Ltd., Shanghai, China) in a 5% CO_2_ incubator at 37 °C with saturated humidity. When the density of cells reached 80–90%, the cells were placed in DMEM high-glucose medium +2% horse serum (GIBCO, Grand Island, NE, USA) and +1% penicillin/streptomycin differentiation medium to induce differentiation. The solution was changed every other day, and the cells became myotube cells after 4 days of differentiation. The myotubes were randomly divided into five groups: control group, model group, CMP50, CMP100, and CMP200 group. Firstly, the cells in the control group and the model group were cultured in differentiation medium for 4 h. Meanwhile, the cells in the CMP 50, 100, and 200 groups were pre-incubated for 4 h in differentiation medium supplemented with 50, 100, and 200 μmol/L CMP, respectively. After 4 h, except for the control group, which continued to be cultured in differentiation medium, the cells in the model group were added to 100 μM H_2_O_2_ in differentiation medium. The cells in the CMP 50, 100, and 200 groups were cultured in differentiation medium including 100 μM H_2_O_2_ and 50, 100, and 200μmol/L CMP, respectively. All of the above groups of cells continued to be cultured for 48 h. The H_2_O_2_ and CMP doses were chosen according to the literature [14,22], and cell cytotoxicity was ruled out by CCK8 assay. Myotubes from each group were either fixed for immunofluorescence staining or collected by trypsinization for biochemical and other studies after 48 h.

### 2.3. Immunofluorescence Analysis

C2C12 cells were cultured in 12-well plates. After differentiation into myotube cells, the cells were pretreated in differentiation medium containing CMP (100 μmol/L) for 4 h, and the cells were further cultured for 48 h with differentiation medium containing 100 μmol/L H_2_O_2_ and CMP100. At the end of the experiment, the myotube cells were stained with immunofluorescence. The cells were first fixed with 4% paraformaldehyde for 15–20 min, washed 3 times with PBS, blocked with blocking solution for 1 h, and then incubated with 5% BSA/5% normal goat serum/PBS overnight with primary antibody. The primary antibody used was rabbit anti-Desmin antibody (ab15200 1:1000, Abcam, Cambridge, MA, USA). The sections were washed in PBS + 0.1% Tween-20 for 5 min each time in two consecutive washes. This was followed by incubation with Alexa Fluor 488 (1:800, Invitrogen, Carlsbad, CA, USA). Imaging was performed on a Leica TCS SP8 (Leica, Mannheim, Germany) confocal microscope. The myotube diameter was the mean value of 50 myotubes in each group, determined using LAS X 3.0 Myotubes containing more than three nuclei were considered to be positive, and the diameters of the myotubes were analyzed using LAS X 3.0.

### 2.4. Myotube Analysis

A total of six areas per well of each group were randomly chosen and their diameters were analyzed. The diameter was observed with the help of LAS X 3.0 by taking the average measurement of each well containing about 50 myotubes that contained a minimum of 3 nuclei. 

### 2.5. Cell Viability Assay

The cell-counting kit-8 (CCK-8) assay (KeyGEN, Nanjing, Jiangsu, China) was performed to evaluate cell viability according to the manufacturer’s protocol. In short, 100 µL/well cells were seeded in 96-well plates. The cells were cultured with the abovementioned method. After cultivation, the cells were collected. After treatment according to the protocol, 10 µL CCK-8 were added to each well and incubated at 37 ° C for 1–4 h. The absorbance per hole was measured at 450 nm with a microplate reader (BMG FLUOstar Omega, Offenburg, Germany).

### 2.6. Flow Cytometry Assay

Cells were cultured in 6-well cell culture plates and treated according to the protocol. For intracellular ROS analysis, the cells were collected and washed once with PBS, and then they were incubated with the 10 µM 2,7-dichlorofluorescein diacetate (DCFH-DA, Beyotime, Shanghai, China) for 20 min at 37 °C. After being washed three times with PBS, the cells were analyzed using a flow cytometer (Beckman Coulter, Brea, CA, USA), and the intracellular ROS levels were expressed as the average DCF fluorescence intensity. For mitochondrial membrane potential (∆Ψm, MMP) analysis, cells were harvested and stained with 500 µL 1× JC-1 dye solution (Beyotime, Shanghai, China) for 20 min at 37 °C in darkness. Then, the cells were washed twice and resuspended by 1× JC-1 staining buffer. The change in fluorescence color was analyzed using flow cytometry (Beckman Coulter, Brea, CA, USA). When MMP is low, JC-1 is present as a monomer and produces green fluorescence, whereas when the inner mitochondrial membrane is highly polarized, JC-1 monomers aggregate to form so-called J-aggregates, which can be observed as red fluorescence. 

### 2.7. Evaluation of Antioxidant Enzyme Activity and Differentiation, Injury, Mitochondrial Dysfunction Markers

The cells were cultured in a 6-well cell culture plate. After differentiation into myotube cells, the cells were pretreated in differentiation medium containing CMP (50/100/200 μmol/L) for 4 h, and the cells were further cultured for 48 h with differentiation medium containing 100 μmol/L hydrogen peroxide and CMP (50/100/200 μmol/L). Then, the supernatant was collected and the contents of antioxidant enzymes CAT and GSH-px in the supernatant of each group were detected with commercial kits (Nanjing Jiancheng Bioengineering Institute, Nanjing, China). CK activity was measured as a differentiation and injury marker using a CK kit (Nanjing Jiancheng Bioengineering Institute, Nanjing, China). 

### 2.8. Evaluation of Cytokines Associated with Sarcopenia

The cells were cultured in 6-well plates. After differentiation into myotube cells, the cells were pretreated in differentiation medium containing CMP (50/100/200 μmol/L) for 4 h, and the cells were further cultured for 48 h with differentiation medium containing 100 μmol/L hydrogen peroxide and CMP (50/100/200 μmol/L). Then, the supernatant of the cells was collected to detect CRP, IL-6, and TNF-α in each group. CRP concentration was determined with an enzyme-linked immunosorbent assay (ELISA) kit (Multisciences, Hangzhou, China) according to the manufacturer’s instructions. The concentrations of IL-6 and TNF-α were measured using commercially available TNF-α- and IL-6-sensitive ELISA (Invitrogen, USA) in accordance with the manufacturer’s instructions.

### 2.9. RNA-seq and Analysis

Total RNA was extracted from C2C12 myotubes with TRIzol reagent (TIANGEN, Beijing, China), and the integrity and total amount of RNA were accurately detected using an Agilent 2100 bioanalyzer, according to the manufacturer’s instructions. Then, the purified RNA was segmented via incubation at 94 °C for 5 min with a fragmentation buffer by Illumina. Then, the first strand of cDNA was synthesized via reverse transcription using random oligo primers. Second-strand synthesis was performed via incubation with RNase H and DNA polymerase I. The purified double-strand cDNA was end-repaired, an a-tail was added, and sequencing joints were connected. cDNA of about 370~420 bp was screened with AMPure XP beads and PCR amplification was performed. PCR products were purified with AMPure XP beads again, and libraries were finally obtained. After the library was constructed, preliminary quantification was performed using a Qubit2.0 fluorometer (Shanghai, China) and the library was diluted to 1.5 ng/uL. Then, an Agilent2100 bioanalyzer (Agilent, Santa Clara, CA, USA) was used to detect the insert size of the library. qRT-PCR could accurately quantify the effective concentration of the library (the effective concentration of the library was greater than 2 nM) and ensure the quality of the library. After the library was qualified, it was sequenced on the Illumina novaseq 6000 platform. The basic principle of sequencing is synthetic sequencing. Four kinds of fluorescently labeled dNTP, DNA polymerase, and joint primers were added to sequenced flow cells for amplification. When each sequencing cluster extended the complementary chain, each fluorescently labeled dNTP was added to release corresponding fluorescence. The sequencer captured the fluorescence signal and converted the optical signal into a sequencing peak through computer software. Thus, the sequence information of the fragment to be tested could be obtained. In the data analysis stage, data quality control was carried out. In order to ensure the reliability of the data analysis, the original data needed to be filtered. This included reads with an adapter, reads containing N (N indicates that base information cannot be determined), and low-quality reads (Qphred ≤ reads, where the base number of 20 accounts for more than 50% of the total read length). At the same time, the contents of Q20, Q30, and GC of clean data were calculated. All subsequent analyses were based on high-quality analysis conducted with clean data. Sequence alignment to the reference genome HISAT2v2.0.5 was used to construct the index of the reference genome, and HISAT2v2.0.5 was used to compare the paired terminal clean reads with the reference genome.

### 2.10. Western Blot Analysis

The C2C12 myotubes were lysed with RIPA buffer (Biosharp, Hefei, China) containing protease inhibitor (Roche, Basel, Switzerland) and phenylmethylsulfonyl fluoride to extract total proteins. The protein was extracted via centrifugation at 12,000× *g* for 15 min at 4 °C, and the protein concentration was determined with a BCA protein assay kit (Thermo Scientific, Waltham, MA, USA). An equal amount of protein (120 µg) was separated by 10–12% SDS-PAGE gel and transferred to the PVDF membranes at different electric currents depending on the size of the protein molecule. The membranes were blocked with 5% non-fat milk and incubated with primary antibodies. Protein expression was detected using a primary antibody. p-Akt (Ser473), p-S6K (Thr389), p-IRS-1 (Thr307), IRS-1, and MuRF1 were from Cell Signaling (Beverly, MA, USA). Akt, S6K, Atrogin-1, and GADPH antibodies were from Proteintech Antibody. PGC-1α and secondary antibodies were from Abcam (Cambridge, MA, USA). 

### 2.11. Statistical Analyses

We used SPSS software version 22 (SPSS Inc., Chicago, IL, USA) for statistical analyses. The values are shown as mean ± standard error of the mean (SEM). Differences among groups were analyzed with a one-way analysis of variance test and LSD methods if the data were homogeneous or the Dunnett’s T3 test if the variance was unequal.

## 3. Results

### 3.1. Effect of CMP on C2C12 Myotube Viability

**Figure 1:**
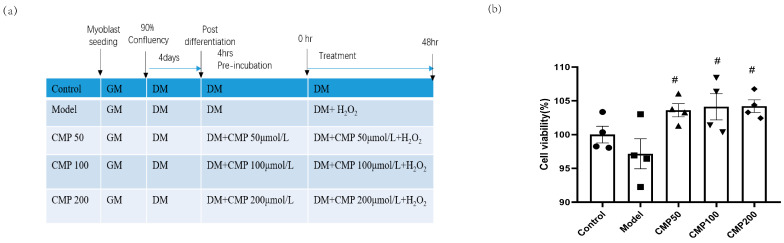
Effect of CMP on C2C12 myotube viability. (**a**) A schematic presentation including different treatments and the course of the experiments. (**b**) Cell viability evaluation of CMP using the CCK-8 assay (n = 4 per group). Control, C2C12 cells with medium control; model, C2C12 cells with 100 um H_2_O_2_ in medium; CMP 50, C2C12 with H_2_O_2_ + 50 umol/L CMP; CMP 100, C2C12 with H_2_O_2_ + 100 umol/L CMP; CMP 200, C2C12 with H_2_O_2_ + 200 umol/L CMP. Error bars indicate SEM. ^#^ *p* < 0.05 versus model group.

We used 100 μmol/L H_2_O_2_ to induce a muscular atrophy cell model in C2C12 myotubes and investigated the effects of CMP on C2C12 myotubes. A schematic presentation including different treatments and the course of the experiments is shown in Figure 1a. In order to assess the effects of CMP on myotube viability, a CCK8 assay was performed. Cell viability was measured in the C2C12 myotubes after treatment with diverse concentrations (50, 100, and 200 μmol/L) of CMP. The cell viability decreased in the model group, whereas it improved with the treatment with CMP (Figure 1b). Compared with the model group, cell viability in the CMP 50, CMP 100, and CMP 200 groups increased by 6.4%, 6.9%, and 7%, respectively. 

### 3.2. Effect of CMP on the Myotube Atrophy in C2C12 Cells

**Figure 2:**
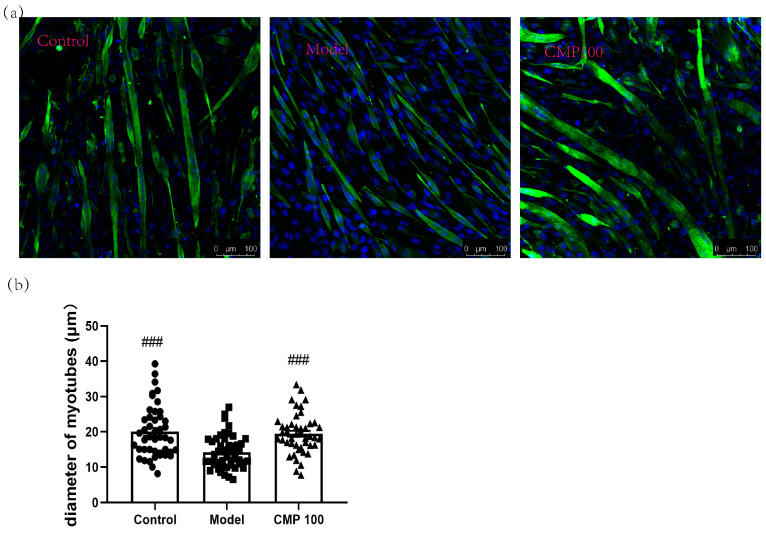
Impact of CMP on the myotube atrophy in C2C12 myotubes. (**a**) Representative images of myotubes. Scale bar = 100 μm. Green indicates Desmin staining, whereas blue indicates DAPI staining of nuclei. (**b**) Average diameters of myotubes. Error bars indicate SEM and are based on three independent experiments (about 50 myotubes per group). All images were captured with identical settings like exposure times and intensity. ^###^ *p* < 0.001 versus model group.

The diameter of myotubes is an important indicator of muscle atrophy. To investigate the effect of CMP on the diameters of the C2C12 myotubes, we conducted immunofluorescence staining for myotubes treated with CMP 100, which was most representative according to the previous results. As shown in Figure 2, the C2C12 myotube diameters significantly decreased in the model group (*p* < 0.001), indicating that muscle atrophy was induced by H_2_O_2_ in C2C12 myotubes. Conversely, the myotube diameters of the CMP100 treatment groups increased significantly (*p* < 0.001) compared with the model group. This indicates that 100 μmol/L CMP could reverse the atrophy of the myotubes.

### 3.3. Effect of CMP on Mitochondrial Potential (JC-1) in C2C12 Cells

**Figure 3:**
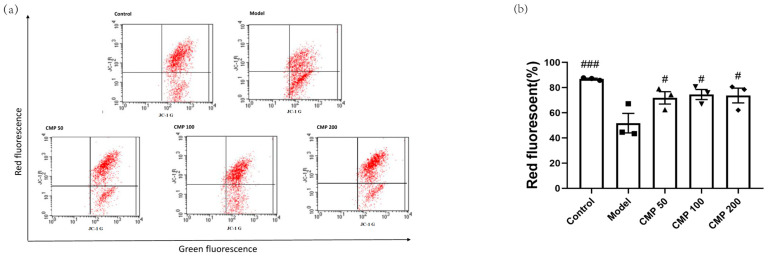
Effect of CMP on the mitochondrial membrane potential. (**a**) Mitochondrial membrane potential was measured through flow cytometry in the control group, model group, and CMP 50, 100, and 200 groups. The x-axis represents green fluorescence intensity, whereas the y-axis indicates red fluorescence intensity. (**b**) The quantitative results were analyzed. The data are expressed as mean ± SEM (n = 3 per group). ^#^ *p* < 0.05, ^###^*p* < 0.001 versus model group.

Decreased MMP is an important marker of mitochondrial damage. One of the most common approaches to monitoring MMP is through the use of JC-1. When the mitochondrial membrane potential is normal, JC-1 is in the aggregation state (showing red fluorescence); when the mitochondrial membrane potential is reduced, JC-1 can be de-aggregated into monomers (green fluorescence), suggesting mitochondrial damage. Therefore, the ratio of aggregates (red fluorescence) to monomers (green fluorescence) reflects the loss of MMP. To explore the effect of CMP on mitochondrial function in myotubes, we performed JC-1 staining. The exogenous addition of H_2_O_2_ led to MMP depolarization in the myotubes. In comparison with the model group, a positive effect of CMP50, CMP100, and CMP200 on mitochondrial membrane potential was observed (*p* < 0.05) (Figure 3b). 

### 3.4. Antioxidant Effect of CMP on C2C12 Myotubes

**Figure 4:**
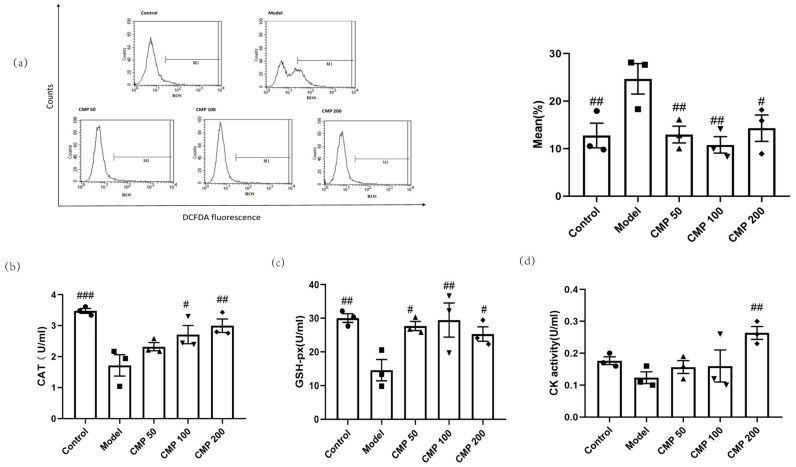
Antioxidant effect of CMP on C2C12 myotubes. (**a**) Effect of CMP on C2C12 myotube ROS production. The left image is the mean fluorescence intensity (MFI) after staining was quantified with flow cytometry in the control group, model group, and low-, medium-, and high-CMP-dose groups, respectively. The right image is a histogram of MFI in each group (n = 3 per group). The x-axis represents DCF intensity, whereas the y-axis indicates the cell count corresponding to fluorescence intensity. (**b**) Effect of CMP on C2C12 myotube catalase activity. (**c**) Effect of CMP on C2C12 myotube glutathione peroxidase activity. (**d**) Effect of CMP on C2C12 myotube creatine kinase activity. Error bars indicate SEM. ^#^ *p* < 0.05, ^##^ *p* < 0.01, ^###^ *p* < 0.001 versus model group.

We also investigated whether CMP had antioxidant abilities. After C2C12 cells were cultured with the method mentioned above, the five groups of cells were collected to test antioxidant ability. In comparison with the control group, enhanced intracellular ROS production in response to the exogenous addition of H_2_O_2_ was observed in the model group, whereas the treatment with CMP significantly decreased intracellular ROS production. As shown in Figure 4a, compared with the model group, the ROS levels in the CMP50 (*p* < 0.01), CMP100 (*p* < 0.01), and CMP200 (*p* < 0.05) groups significantly decreased. This suggests that CMP could significantly reduce ROS production. Compared with the model group, CAT activities in the CMP 100 (*p* < 0.05), CMP 200 (*p* < 0.01), and control groups (*p* < 0.001) significantly increased (Figure 4b). GSH-Px activities in the CMP 50 (*p* < 0.05), CMP100 (*p* < 0.01), and CMP200 (*p* < 0.05) groups significantly increased compared to the model group (Figure 4c). CK activities in the CMP200 group significantly increased compared to the model group (*p* < 0.01) (Figure 4d). This shows that CMP reduced oxidative stress levels of C2C12 cells induced by H_2_O_2_.

### 3.5. Effect of CMP on Cytokines Associated with Sarcopenia

**Figure 5:**
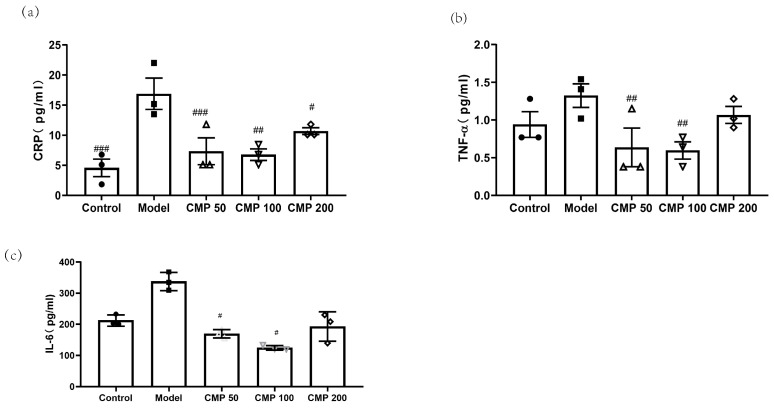
Effect of CMP on cytokines associated with sarcopenia. (**a**) Effect of CMP on CRP concentration in C2C12 myotube supernatant. (**b**) Effect of CMP on TNF-α concentration in C2C12 myotube supernatant. (**c**) Effect of CMP on IL-6 concentration in C2C12 myotube supernatant. Error bars indicate SEM. ^#^ *p* < 0.05, ^##^*p* < 0.01, ^###^*p* < 0.001 versus model group.

To investigate the effect of CMP on cytokines associated with sarcopenia in C2C12 myotubes, after C2C12 cells were cultured with the method mentioned above, cells were collected. Then, the levels of CRP, TNF-α, and IL-6 in the myotubes were measured using an ELISA kit. As shown in Figure 5a, compared with the model group, the concentration of CRP in the CMP 50 (*p* < 0.001), CMP 100 (*p* < 0.01), CMP 200 (*p* < 0.05), and control groups (*p* < 0.001) was significantly lower. The level of TNF-α was also decreased in the CMP 50/100 groups (*p* < 0.01) (Figure 5b). The secretion of IL-6 was significantly decreased in the CMP 50/100 groups (*p* < 0.05) compared with the model group (Figure 5c). The results suggest that CMP could decrease inflammation levels associated with sarcopenia.

### 3.6. RNA-seq Analysis

**Figure 6:**
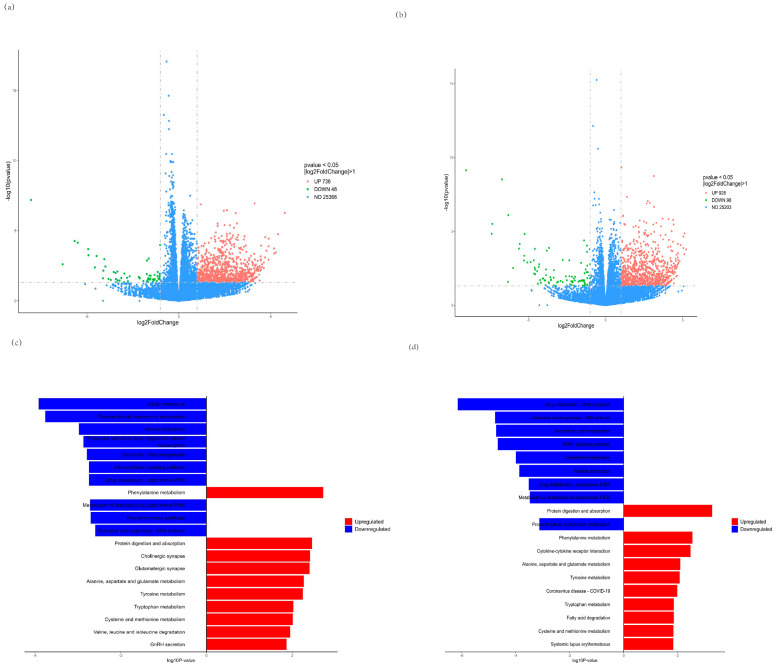
Effect of CMP on RNA−seq. (**a**,**b**) Volcano plot showing differentially expressed genes (DEG) in the CMP vs. model group and the CMP vs. control group, respectively (n = 5 per group). Quantitative meta-analysis results with a cut-off of 0.05 for *p*-value and 2 for fold-change. (**c**,**d**) Kyoto Encyclopedia of Genes (KEGG) pathway enrichment analysis revealed that these genes were associated with signaling pathways related to downregulated insulin resistance and upregulated protein digestion and absorption in the CMP vs. model group (**c**). Protein digestion and absorption was significantly upregulated in the CMP vs control group (**d**). DEG, differentially expressed genes; KEGG, Kyoto Encyclopedia of Genes.

We further investigated global transcriptome changes after treatment with CMP for C2C12 myotubes by RNA-seq in the CMP 100 vs. model group and the CMP 100 vs. control group. The RNA sequencing results of each group were analyzed, and the genes with |log2foldchange| > 1 and *p* < 0.05 were selected for statistical analysis. As is shown in the volcano plot (Figure 6a,b), a total of 784 differentially expressed genes (DEGs), including 48 downregulated and 736 upregulated genes, were identified in the CMP vs. model group (Supplementary Tables S1 and S2). A total of 1024 DEGs, including 98 downregulated and 98 upregulated genes, were identified in the CMP vs. control group. Moreover, Kyoto Encyclopedia of Genes (KEGG) enrichment analysis of the DEGs was performed. Notably, genes that showed downregulated expression were enriched in insulin resistance and genes that upregulated expression were enriched in protein digestion and absorption the in CMP vs. model group (Figure 6c), suggesting that the CMP might relieve insulin resistance and promote protein synthesis. KEGG enrichment analysis of the DEGs in the CMP vs. control group also showed that protein digestion and absorption was significantly upregulated (Figure 6d).

### 3.7. Effect of CMP on the Protein Expressions of IRS−1, Akt, S6K, IRS1, MuRF1, Atrogin−1, and PGC−1α

**Figure 7:**
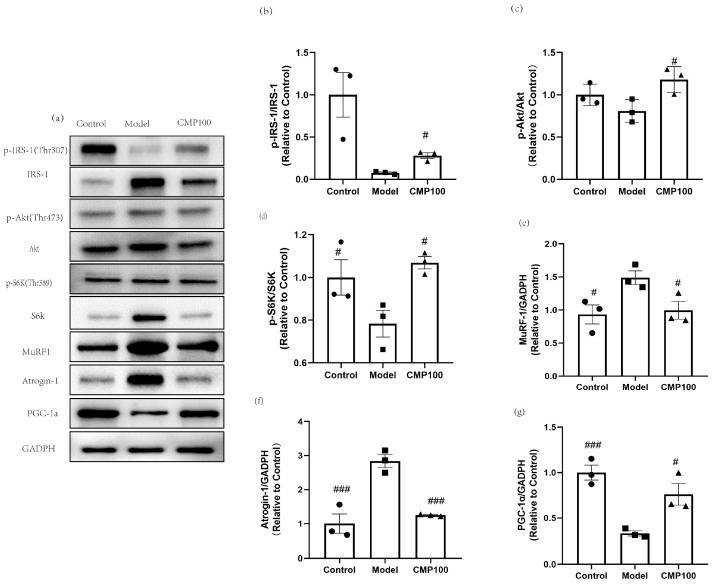
Regulation of CMP on the sarcopenia-related proteins in C2C12 myotubes. (**a**) Related proteins were analyzed via Western blot. (**b**) Effect of CMP 100 on p-IRS-1/IRS-1 in C2C12 myotubes. (**c**) Effect of CMP 100 on p-Akt/Akt in C2C12 myotubes. (**d**) Effect of CMP 100 on p-S6K/S6K in C2C12 myotubes. (**e**) Effect of CMP 100 on MuRF1 in C2C12 myotubes. (**f**) Effect of CMP 100 on Atrogin-1 in C2C12 myotubes. (**g**) Effect of CMP 100 on PGC-1α in C2C12 myotubes. n = 3, Error bars indicate SEM. ^#^ *p* < 0.05 versus model group, ^###^ *p* < 0.001 versus model group.

In order to further evaluate the effect of CMP on the protein expression of H_2_O_2_-induced muscular atrophy in the insulin resistance pathway and the protein synthesis signaling pathway, sarcopenia-related proteins after treatment with CMP were detected by Western blots (Figure 7a). CMP100 was chosen because the medium dose was more representative based on the previous results. In order to explore the effect of CMP on insulin resistance, insulin signaling-related proteins were detected, including p-IRS−1, IRS−1, Akt, and p-Akt. As is shown in Figure 7b,c, the addition of CMP100 increased the phosphorylation of IRS-1 and Akt (*p* < 0.05). Akt/mTOR signaling is considered to be one of the key pathways in muscle protein synthesis [33]. Therefore, we examined the phosphorylation status of S6K, which is a critical downstream gene of mTOR. As shown in Figure 7d, the addition of CMP100 increased the phosphorylation of S6K (*p* < 0.05). In addition, since MuRF1 and Atrogin-1 were used as important markers of muscle atrophy, we also detected these two proteins’ expressions. As is shown in Figure 7e,f, CMP could significantly decrease protein expressions of MuRF1 and Atrogin-1. PGC-1α plays an important role in mitochondrial biogenesis. In the present study, we found that CMP could increase PGC-1α expression (Figure 7g). These results further confirm that CMP could improve mitochondrial dysfunction, activate the IRS-1/Akt/p70s6k signaling pathway, and promote protein synthesis, thereby reducing muscle atrophy.

## 4. Discussion

In the present study, to better simulate skeletal muscle aging caused by mitochondrial dysfunction and oxidative stress, a C2C12 muscular atrophy model was induced via H_2_O_2_. A previous study showed that 100 μmol/L H_2_O_2_ treatment of myotubes could cause muscle atrophy [34]. Consistent conclusions were also obtained in this study, which shows that 100 μmol/L H_2_O_2_ significantly promoted the atrophy of myotubes, which indicates the successful establishment of our muscle atrophy model. Our research suggests that CMP might be an effective nutritional intervention for sarcopenia. The results showed that CMP could significantly increase the diameter of C2C12 myotubes. We also found that CMP could improve mitochondrial dysfunction and antioxidants and reduce inflammatory cytokines levels associated with sarcopenia. RNA-seq analysis showed that CMP could relieve insulin resistance and promote protein digestion and absorption, which indicates that CMP might promote protein synthesis and improve muscle atrophy. The Western blot analysis further confirmed that CMP could promote the activation of the IRS1/Akt/S6K protein synthesis signaling pathway and reduce insulin resistance. In addition, CMP could decrease the expression of MuRF1 and Atrogin-1, which are important markers of muscle atrophy. Furthermore, CMP could increase PGC-1α expression, which is one of the most important indicators of mitochondrial biogenesis. All the above results prove that CMP has the potential to improve muscular atrophy. 

The molecular mechanism of sarcopenia has not been fully understood and is still being actively explored. Current studies on the pathogenesis of sarcopenia mainly include the imbalance of skeletal muscle protein synthesis and decomposition, inflammatory factors and insulin resistance, oxidative stress and mitochondrial dysfunction, the imbalance of dynamic regulation of cytokines, changes in hormone levels, motor neuron degeneration, genes and genetics, etc., and these factors interact with each other, resulting in varying degrees of muscle mass and/or muscle strength decline. Eventually, sarcopenia occurs [35]. Increased oxidative stress and inflammation and decreased mitochondrial function are thought to be important upstream signals of skeletal muscle atrophy caused by various diseases [36,37]. In particular, mitochondrial dysfunction is thought to be a major factor in age-related muscle degeneration [37]. The increase in ROS, which controls the redox signaling pathway of muscle fibers, and the substantial decrease in the number and function of skeletal muscle mitochondria, will reduce skeletal muscle protein synthesis and promote protein hydrolysis [22]. Mitochondria is the main source of ROS in muscle tissue. The increase in ROS production may be related to the decreased function of electron transport during aging. Many studies have shown that mitochondrial dysfunction is one of the most important features of sarcopenia [38,39]. In the present study, we found that CMP could significantly reduce the ROS production of C2C12 myotubes. In addition, the mitochondrial membrane potential of amyotrophic cells increased significantly after CMP treatment. PGC-1α plays an important role in mitochondrial biogenesis [40]. Studies show that decreased mitochondrial biogenesis is closely related to sarcopenia [41]. In our study, we found that CMP could promote the protein expression of PGC-1α and improve mitochondrial dysfunction. We also evaluated the effect of CMP on antioxidant enzyme activity. After CMP treatment, the activities of CAT, GSH-Px, and CK significantly increased. These results indicate that CMP could improve mitochondrial dysfunction and reduce oxidative stress. Studies have shown that several cytokines (TNF-α, CRP, and IL-6) play an important role in the pathogenesis of sarcopenia [42,43,44,45]. The high level of TNF-α expression in vivo could inhibit the Akt/mTOR pathway and led to the increase of muscle protein catabolism, and also activated the ubiquitin–proteasome pathway to promote muscle proteolysis [24]. In addition, the phosphorylation of S6K1 was decreased in IL-6-infused muscles [25]. We found that CMP could reduce the levels of TNF-α, CRP, and IL-6 in our study and inhibit the Akt/mTOR pathway. 

Mitochondrial dysfunction and inflammation can also cause insulin resistance [46]. Insulin resistance is related to the loss of lean mass in older men [47]. Insulin resistance is one of the pathogeneses of sarcopenia. Since insulin activates the PI3K/Akt/ mTOR pathway analogously to IGF-1, insulin resistance plays an important role in inhibiting the activation of the Akt/ mTOR protein synthesis signaling pathway in muscle atrophy [13]. In our study, RNA-seq analysis showed that CMP could significantly downregulate the insulin resistance signaling pathway. The Western blot analysis further confirmed that CMP could promote the activation of the IRS1/Akt/S6K protein synthesis signaling pathway and decrease the expression of MuRF1 and Atrogin-1. Akt/mTOR signaling is considered to be one of the key pathways in muscle protein synthesis [33]. mTOR is one of the target molecules of Akt, which can phosphorylate and activate P70S6K. Activated P70S6K can make ribosomal S6 protein in a state of high-energy phosphorylation, enhance mRNA translation, and thus promote protein biosynthesis [16]. In the present study, we found that CMP100 activated IRS-1, Akt, and p70S6K signaling molecules with more elevated phosphorylation compared to the model group.

**Figure 8:**
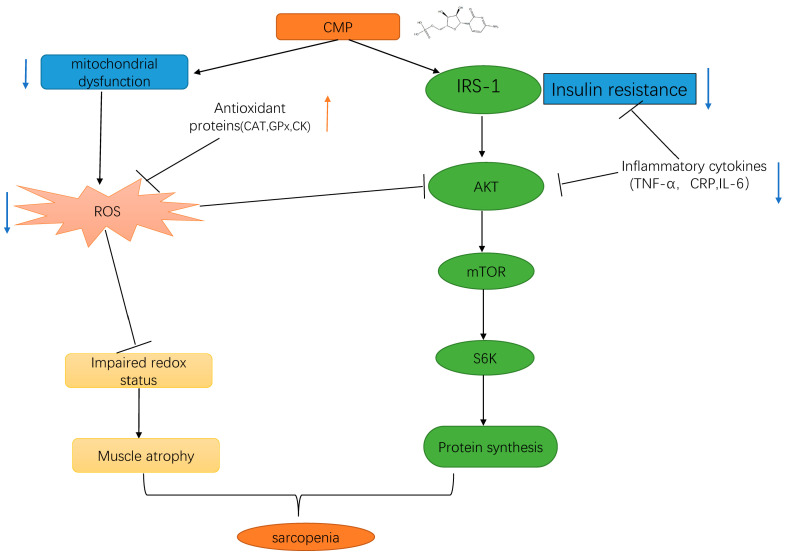
Schematic representation of the results of the present study. We found that CMP improved H_2_O_2_-induced atrophy in C2C12 myotubes by activating the IRS-1/Akt/S6K pathway. Up-regulation are represented by orange arrowheads; Down-regulation are represented by blue arrowheads; CMP, cytidine 5′-monophosphate.

Overall, the present study provides the first evidence for the role of CMP in H_2_O_2_-induced C2C12 muscular atrophy cells, and the underlying mechanism may be associated with activating the IRS-1/Akt/S6K pathway to promote protein synthesis by reducing insulin resistance and mitochondrial dysfunction (summarized in Figure 8). Our study provides a theoretical basis for the application of CMP in the treatment of sarcopenia. There are several limitations in our study. Firstly, this study was performed under a single atrophying condition. Secondly, this study was based on artificially differentiated myotubes from a transformed cell line. Therefore, more conditions or cell models warrant further investigation. The inhibitory effect of CMP on muscle atrophy has not been demonstrated in animals or humans. Further studies are needed to confirm the effects of CMP on sarcopenia in animals or humans. 

## 5. Conclusions

In conclusion, we are the first to demonstrate the protective role of CMP and its potential mechanism of protection against muscle atrophy in C2C12-differentiated myotubes. In the present study, immunofluorescence analysis showed that CMP significantly increased the diameter of C2C12 myotubes. We found that CMP could improve mitochondrial dysfunction and antioxidants and reduce cytokines levels associated with sarcopenia. RNA-seq analysis showed that CMP could relieve insulin resistance. The Western blot analysis further confirmed that CMP could promote the activation of the IRS-1/Akt/S6K protein synthesis signaling pathway and decrease the expression of markers of muscle atrophy MuRF1 and Atrogin-1. The above results suggest that CMP protects myotubes from H_2_O_2_-induced atrophy and that its mechanism is associated with activating the IRS-1/Akt/S6K pathway to promote the protein synthesis. Thus, this suggests that CMP supplementation is an effective and optimal strategy for sarcopenia prevention and treatment.

## Data Availability

The data presented in this study are available on request from the corresponding author.

## Supplementary Materials

The following supporting information can be downloaded at: https://www.mdpi.com/article/10.3390/antiox13020249/s1. Table S1: The down-regulated DEGs in CMP vs. Model; Table S2: The Up-regulated DEGs in CMP vs. Model.
